# Expanding the chemical space for natural products by *Aspergillus*-*Streptomyces* co-cultivation and biotransformation

**DOI:** 10.1038/srep10868

**Published:** 2015-06-04

**Authors:** Changsheng Wu, Boris Zacchetti, Arthur F.J. Ram, Gilles P. van Wezel, Dennis Claessen, Young Hae Choi

**Affiliations:** 1 Molecular Biotechnology, Institute of Biology, Leiden University, Sylviusweg 72, 2333 BE, The Netherlands; 2 Natural Products Laboratory, Institute of Biology, Leiden University, Sylviusweg 72, 2333 BE, The Netherlands

## Abstract

Actinomycetes and filamentous fungi produce a wide range of bioactive compounds, with applications as antimicrobials, anticancer agents or agrochemicals. Their genomes contain a far larger number of gene clusters for natural products than originally anticipated, and novel approaches are required to exploit this potential reservoir of new drugs. Here, we show that co-cultivation of the filamentous model microbes *Streptomyces coelicolor* and *Aspergillus niger* has a major impact on their secondary metabolism. NMR-based metabolomics combined with multivariate data analysis revealed several compounds that correlated specifically to co-cultures, including the cyclic dipeptide cyclo(Phe-Phe) and 2-hydroxyphenylacetic acid, both of which were produced by *A. niger* in response to *S. coelicolor*. Furthermore, biotransformation studies with *o*-coumaric acid and caffeic acid resulted in the production of the novel compounds (*E*)-2-(3-hydroxyprop-1-en-1-yl)-phenol and (2*E*,4*E*)-3-(2-carboxy-1-hydroxyethyl)-2,4-hexadienedioxic acid, respectively. This highlights the utility of microbial co-cultivation combined with NMR-based metabolomics as an efficient pipeline for the discovery of novel natural products.

Filamentous microorganisms have an enormous potential for the production of a wide range of bioactive compounds, with application as among others antibiotic, anticancer or antifungal drugs^1^,^2^,^3^. In particular, the emergence of infectious diseases involving multi-drug resistant (MDR) bacterial pathogens since the 1980s has reinforced the screening efforts using filamentous microorganisms. It is likely that in terms of the biosynthetic potential of microbes we have only seen the tip of the iceberg^4^. However, the return of investment on high-throughput screening campaigns has dropped alarmingly, resulting in an almost complete withdrawal of BigPharma from the antibiotic scene^5^. Genome sequencing has now revealed that prolific antibiotic producers, and in particular fungi^6^,^7^,^8^ and actinomycetes^9^,^10^,^11^, which had been intensively studied for decades, harbour far more biosynthetic gene clusters for natural products than originally anticipated. The challenge scientists now face is to elicit the expression of these potential treasures, via chemical, genetic or biological triggers^12^,^13^. Strategies that have been employed include heterologous expression^14^, screening or selecting for antibiotic resistant strains^15^,^16^, and the use of chemical elicitors^17^,^18^. Besides the activation of cryptic gene clusters in known species, a second major reservoir for natural products is formed by microorganisms that have not yet been cultivated, which recently led to the discovery of novel antibiotics in unexpected microbial sources^19^,^20^.

Growth in microbial communities or interactions between different microorganisms is the next logical step in the search for new molecules, as many natural products can be expected to be activated specifically by competitors or symbionts in the natural habitat. Microbial co-cultures have been shown to elicit the biosynthesis of novel metabolites, thus increasing the pre-existent chemical diversity^21^,^22^. The model microorganisms that are studied intensively in our laboratory are *Streptomyces coelicolor*, a Gram-positive bacterium with a complex multicellular lifestyle^23^, and the fungus *Aspergillus niger*. It was shown previously that interaction with bacteria may have profound effects on the producing ability of *Aspergillus* species^24^,^25^. Here, we report the application of co-cultivation of *S. coelicolor* and *A. niger* for the discovery of novel natural products. In addition, the structurally related *o*-coumaric acid and caffeic acid were specifically converted into the novel molecules (*E*)-2-(3-hydroxyprop-1-en-1-yl)-phenol and (2*E*,4*E*)-3-(2-carboxy-1-hydroxyethyl)-2,4-hexadienedioxic acid, respectively. We thereby apply NMR-based metabolomics, a technology that is particularly effective in identifying molecules in complex biological mixtures, which therefore facilitates the direct biochemical analysis of the community metabolism^26^. While routinely applied in plant metabolomics, its application in microbial research is rare^27^.The combination of microbial co-cultivation with NMR-based metabolomics is presented as an efficient way to discover new molecules.

## Results and discussion

**Figure 1:**
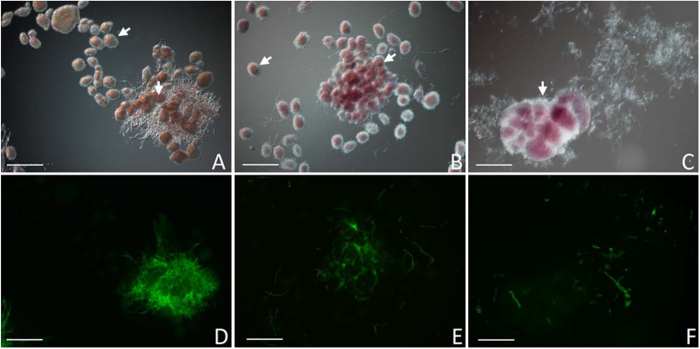
Mycelial interactions between *Aspergillus niger* AR19#1 (large open mycelial structures) and *Streptomyces coelicolor* A3(2) M145 (small red-pigmented pellets indicated by arrow heads) during co-culture. Strain AR19#1 is a derivative of *A. niger* N402 which expresses eGFP. Samples were observed following inoculation of *A. niger* at 24 h (**A**,**D**), 72 h (**B**,**E**), and 96 h (**C**,**F**). The reduction in fluorescence intensity in the eGFP-positive hyphae of *A. niger* (**D**-**F**) highlights the strong decline in viable fungal biomass in later stages of the co-culture. Bar, 250 μm (**A**,**B**) or 125 μm (**C**).

The interaction between the two filamentous model microbes *Streptomyces coelicolor* A3(2) M145 and *Aspergillus niger* N402 was studied in co-cultivations in submerged NMMP cultures. Preliminary experiments indicated that the pH of the medium had contrasting effects on the growth of both microbes: whereas *Streptomyces* species grow well at neutral pH, those of *Aspergillus* grow optimally at lower pH values. A phosphate buffer of pH 5 was optimal for obtaining sufficient biomass of both microorganisms for a mutual interaction to occur. As the growth rate of *A. niger* was higher under these conditions, the fungal spores were added to the culture flasks 72 h after inoculation of *S. coelicolor*. To allow the ready visualization of the interaction between the two microorganisms, we used a test setup with *A. niger* AR19#1 which expresses eGFP^28^. *A. nige*r AR19#1 formed pellets with a diameter of 100-500 μm that were rapidly colonized by *S. coelicolor* within 24 h after addition of *A. niger* to the *Streptomyces* culture. The colonization initiated with the adhesion of *S. coelicolor* to the *A. niger* biomass (Fig. 1A,D), eventually leading to pellets that were largely covered with *Streptomyces* biomass after 72 h of co-cultivation (Fig. 1B,E). Notably, loose fungal hyphae were evident in the culture broth in the following 24 h, inferring that the fungal mycelium was almost completely degraded by the bacterium (Fig. 1C,F).

**Figure 2:**
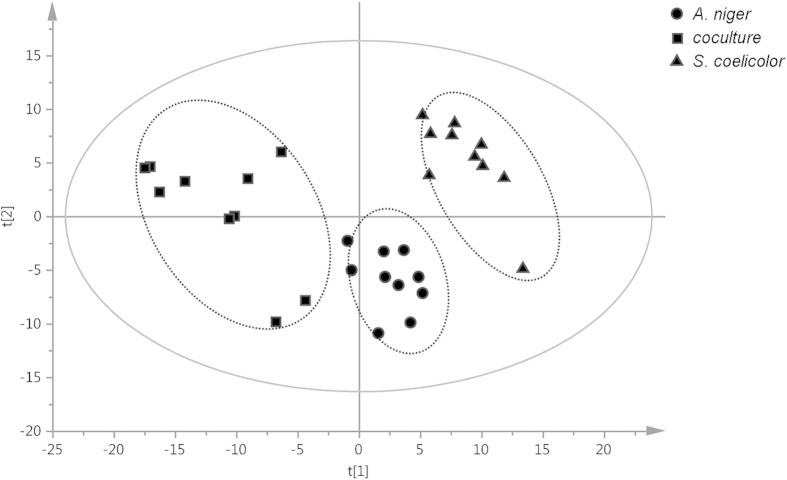
Unsupervised (PCA) multivariate data analysis of the ^1^H NMR fingerprint data included in Figure S1. Circles represent the *S. coelicolor* monoculture, squares the *A. niger* monoculture, while the co-culture of *S. coelicolor* and *A. niger* is represented by triangles.

**Figure 3:**
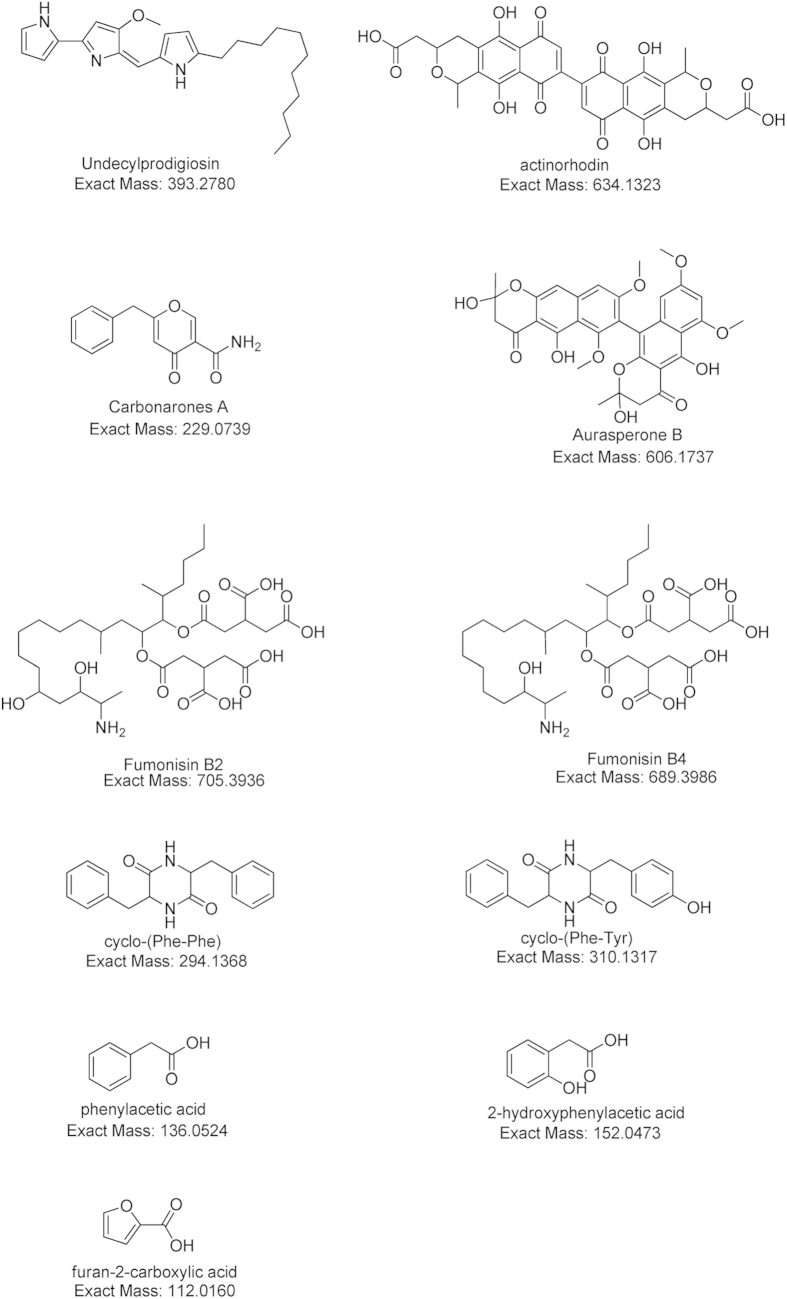
Major discriminating compounds responsible for the PCA separation (Fig. 2) of the *S. coelicolor* and *A. niger* monocultures from their co-culture. *S. coelicolor* monoculture: undecylprodigiosin and actinorhodin; *A. niger* monoculture: carbonarones A, aurasperone B, fumonisin B2 and fumonisin B4; Co-culture: cyclo(Phe-Phe), cyclo(Phe-Tyr), phenylacetic acid, 2-hydroxyphenylacetic acid, and furan-2-carboxylic acid. The shown theoretical exact mass of each compound was calculated with *ChemDraw Ultra 12.0* software. The structure elucidation was done on the basis of NMR and/or experimental UHPLC-TOF-MS high resolution mass, and the spectral data assignments were summarized in Table 1.

Previous studies have shown that evident metabolic changes occur when different organisms are grown together^29^. An NMR-based metabolomics approach was employed to study such effects in the *Aspergillus-Streptomyces* co-cultivations, using *S. coelicolor* A3(2) M145 and *A. niger* N402 (the plasmid-free parent of strain AR19#1, i.e. not expressing eGFP). The method of NMR-based metabolomics was performed according to our previously published protocol, including sample preparation, NMR analysis, metabolite identification and multivariate data analysis^26^. In the current study, a total of 30 liquid cultures (10 for each monoculture and 10 for the coculture) were separately extracted followed by global NMR-profiling of the secondary metabolites (Figure S1). The ^1^H NMR spectra were then subjected to statistical analysis. An unsupervised principal component analysis (PCA) revealed clustering of the samples in three groups, indicating that the co-culture fingerprints did not overlap with the two corresponding monoculture clusters (Fig. 2). This showed that the dataset contained information that allowed the discrimination of the chemical composition of the co-culture from that of the monocultures. Features exclusively related to the co-culture were found within the dataset of induced compounds (see below), implying that microbial interactions modulated the biosynthetic pathways for the production of secondary metabolites. The PCA loading plot (not shown) allowed detecting biomarkers responsible for the discrimination among the three groups. The compounds were identified with different NMR techniques (1D and/or 2D experiments) and UHPLC-TOF-MS analysis (positive and/or negative modes) (Table 1) and the results were compared with spectroscopic data from literature. The major discriminators for the *S. coelicolor* culture were the well-studied pigmented antibiotics undecylprodigiosin and actinorhodin^30^,^31^, while the *A. niger* monoculture was abundant in the γ-pyrone derivative carbonarone A^32^, as well as several minor components, namely naphtho-γ-pyrone aurasperone B, and fumonisins B_2_ and B_4_^33^,^34^. Notably, some compounds accounting for the PCA separation of the metabolites in the co-culture were identified as the previously described compounds cyclo-(Phe-Phe)^35^, cyclo-(Phe-Tyr)^36^, phenylacetic acid^37^, 2-hydroxyphenylacetic acid^37^, and furan-2-carboxylic acid^38^ (Fig. 3).

Recently, the implementation of the co-cultivation strategy attracted considerable interest and application, since it proved to be an effective way to harvest unique structures with pronounced biological activities^29^. It may trigger biosynthetic pathways for ‘cryptic’ natural products that would otherwise remain silent under standard laboratory culture conditions. However, often it has not been established which of the co-cultivated microorganisms is actually responsible for manufacturing these metabolites. Here, we focused on the production of cyclo(Phe-Phe) that was produced abundantly in the co-culture of *A. niger* with *S. coelicolor*. Since Aspergilli produce 2,5-diketopiperazine-type compounds^29^,^39^, *A. niger* was the most obvious candidate. To test this hypothesis, *A. niger* was inoculated into the culture filtrate (supernatant without bacterial biomass) of a 5-day-old *S. coelicolor* culture and incubated for another two days. ^1^H NMR metabolic profiling (Figure S2) unambiguously demonstrated that the culture filtrate of *S. coelicolor* effectively elicited the production of cyclo(Phe-Phe) and phenylacetic acid by *A. niger*. We hypothesize that *A. niger* metabolizes phenylalanine into phenylacetic acid, which is then further oxidized to 2-hydroxyphenylacetic acid^40^, while cyclo(Phe-Phe) can be synthesized by condensation and cyclization of two molecules of phenylalanine^41^,^42^.

**Figure 4:**
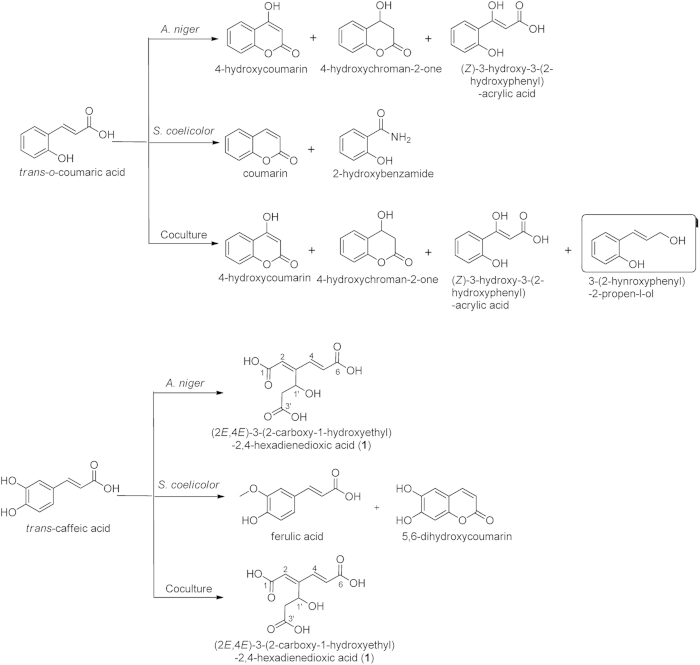
Biotransformation products of o-coumaric acid and caffeic acid by S. *coelicolor* and *A. niger* monocultures and their co-culture. The boxed compound (*E*)-2-(3-hydroxyprop-1-en-1-yl)-phenol was exclusively detected in co-culture. The structurally novel molecule (2*E*,4*E*)-3-(2-carboxy-1-hydroxyethyl)-2,4-hexadienedioxic acid was derived from caffeic acid biotransformation by fungus, while conversion rate was around 3.5 times in co-culture

Thus, compounds in the culture fluid derived from *S. coelicolor* allowed the production of secondary metabolites by *A. niger*, without the need for physical interaction between them. In contrast, direct contact was required for the biosynthesis of the aromatic polyketides orsellinic acid, lecanoric acid, F-9775A and F-9775B in a co-culture of *Aspergillus nidulans* and *Streptomyces hygroscopicus*^43^. We then investigated whether exogenously added compounds could also act as starter material for bioconversion to new compounds. For this, co-cultivation was done in the presence of one of a variety of hydroxycinnamic acids. Seven structurally related molecules, namely cinnamic acid, *o-*, *m-* and *p*- coumaric acid, caffeic acid, ferulic acid, and sinapinic acid, were fed as substrates to the co-culture and to each of the monocultures. After incubation, the cultures were extracted with ethyl acetate and subsequently subjected to ^1^H NMR and UHPLC-TOF-MS analysis (positive and/or negative modes) without further chromatographic fractionation. All of the compounds obtained by bioconversion by the three types of cultures were unambiguously identified on the basis of spectral data (Table S1-S7) and summarized (Figure S3-S9). All structures could be readily elucidated based on spectra of known compounds, except for the novel compound **1** (see below). Under these culturing conditions, the majority of substrates were almost completely consumed by *A. niger*, whereas *S. coelicolor* exhibited a limited capacity to convert these substrates, yielding at best trace amounts of biotransformation products. However, although the biotransformation process in co-cultures was dominated by *A. niger*, both qualitative and quantitative differences were found between the mono- and co-cultures in terms of the biotransformation compounds. The addition of *o*-coumaric acid to the co-culture yielded (*E*)-2-(3-hydroxyprop-1-en-1-yl)-phenol, which was not produced in either of the two monocultures (Fig. 4).

Unexpectedly, caffeic acid was converted by *A. niger* in both mono- and co-culture into a previously undescribed compound (**1**). Its molecular formula, C_9_H_10_O_7,_ was determined using UHPLC-TOF-2Q high-resolution mass analysis with an ion at *m/z* 211.0254 [M – H_2_O – H]^–^ (calculated mass for C_9_H_8_O_6_ is 211.0254). The ^1^H NMR spectrum exhibited a characteristic signal corresponding to a *trans-*substituted olefinic bond, *δ*_H_ 6.42 (d, *J* = 16.2 Hz), 7.47 (dt, *J* = 16.2, 0.6 Hz), which indicated the acrylic acid side chain of caffeic acid resisted the biodegradation. The disappearance of the typical ABX system proton signals for 1,2,4-trisubstituted benzene moiety implied that this aromatic ring had been opened. With the aid of 2D NMR experiments (^1^H-^1^H COSY, HSQC, HMBC, Figure S10-S14), the planar structure of compound **1** was unambiguously identified as (2*E*,4*E*)-3-(2-carboxy-1-hydroxyethyl)-2,4-hexadienedioxic acid (Table 2) shown in Fig. 1. The formation of **1** was likely due to oxygenation of caffeic acid resulting in cleavage of the aromatic ring between two adjacent hydroxyl groups, thus producing two carboxyl groups at the site of ring opening^44^. We propose that the resulting product 4-carboxymethylene-2,5-heptadienoic acid^44^ reacts with one molecule of water to generate the final adduct (2*E*,4*E*)-3-(2-carboxy-1-hydroxyethyl)-2,4-hexadienedioxic acid (Figure S7). In view of the oxidative cleavage of the benzene ring, the geometry at theΔ^2^,^3^ double bond was established as *E*. Because the nucleophilic addition of olefin with H_2_O tends to generate racemates, the stereochemistry at C-1’ was assigned to be both *R* and *S*. The (2*E*,4*E*)-3-(2-carboxy-1-hydroxyethyl)-2,4-hexadienedioxic acid bioconversion rate was accelerated when *A. niger* was co-cultivated with *S. coelicolor*, to around 3.5 times the rate of that occurring in the fungal monoculture.

In conclusion, co-cultivation of *S. coelicolor* and *A. niger* allowed the production of the compounds cyclo(Phe-Phe), 2-hydroxyacetic acid and phenylacetic acid by *A. niger* that were not produced by its monoculture. The fact that the bacterial cell-free filtrate was sufficient for cyclo(Phe-Phe) and phenylacetic acid production by *A. niger* proves that the change in fungal phenylalanine metabolism was induced by secreted compounds that act as starter molecules or as elicitors of silent biosynthetic pathways, rather than by cell-to-cell contact with or nutrient depletion by *S. coelico*lor. NMR-based metabolomics coupled to eliciting compounds by microbial co-cultivation is thereby a very effective strategy to dereplicate known molecules and effectively highlighted the compounds induced specifically by co-cultivation. In addition, chemical modification of hydroxycinnamic acids was achieved by *Aspergillus* and/or *Streptomyces* biotransformation, whereby only the co-culture specifically converted *o*-coumaric acid into (*E*)-2-(3-hydroxyprop-1-en-1-yl)-phenol. Importantly, besides novel metabolic activity, the biotransformation also allowed the discovery of the novel compound (2*E*,4*E*)-3-(2-carboxy-1-hydroxyethyl)-2,4-hexadienedioxic acid, which was produced following addition of caffeic acid. Thus, implementation of co-cultivation and biotransformation has the capacity to increase chemical diversity, which offers new leads for drug-discovery efforts.

## Methods

### Bacterial strains and culturing conditions

*Streptomyces coelicolor* A3(2) M145^45^ was obtained from the John Innes Centre strain collection, *Aspergillus niger* N402 (*cspA1*) is a derivative of *A. niger* ATCC9029. *A. niger* AR19#1^28^ expresses eGFP under control of the *A. nidulans gpdA* promoter. NMMP^45^ was used as liquid minimal media for all co-cultivation experiments. Growth curves of *S. coelicolor* M145 were set up in 50 ml NMMP with glucose (0.5% w/v) as described^46^, with an inoculum of 10^6^ spores in 250 ml flasks equipped with a spring, and grown at 30 °C with constant shaking at 160 rpm. After 72 h, 10^5^ spores of *A. niger* N402 were added to the culture, and cultivation continued for another 72 h. In parallel, monocultures of *S. coelicolor* and *A. niger* were grown for 144 h and 72 h, respectively, corresponding to the cultivation time of each strain in the co-culture. Ten replicates of each experiment, namely *Aspergillus niger* monoculture, *Streptomyces coelicolor* monoculture, and the coculture (so 30 samples in total), were prepared for the metabolomics studies. Fluorescence and corresponding light micrographs were obtained with a Zeiss Axioscope A1 upright fluorescence microscope (with an Axiocam Mrc5 camera at a resolution of 37.5 nm/pixel) as described^47^. The green fluorescent images were created using 470/40 nm band pass (bp) excitation and 525/50 bp detection.

### Biotransformation of hydroxycinnamic acids

Seven hydroxycinnamic acid congeners (cinnamic acid, *o*-coumaric acid, *m*-coumaric acid, *p*-coumaric acid, caffeic acid, ferulic acid, and sinapinic acid) were selected as substrates for the biotransformation study. For this, 10 mg of each substrate dissolved in 200 μl DMSO were added to the co-culture, 24 h after addition of *A. niger* to the *S. coelicolor* culture. In parallel, the substrates were added to monocultures of *S. coelicolor* (after 96 h) and *A. niger* (after 24 h), corresponding to the cultivation time of each strain in the co-culture. The biotransformation products were analyzed 48 h after adding the substrates. All biotransformation experiments were conducted in triplicate.

### Extraction of metabolites

Culture replicates were harvested by centrifugation at 4000 rpm for 10 min. The culture broth (50 ml) was extracted twice with 20 ml of ethyl acetate. The organic phase was washed with 30 ml of water and subsequently dried with 5 g of anhydrous Na_2_SO_4_. Finally, the EtOAc was removed under vacuum at 38 °C and the residue was dissolved in 2.0 ml of EtOAc in a microtube (Eppendorf type-5415C, Hamburg, Germany). The solvent was then evaporated at room temperature under nitrogen gas, and subsequently dipped into liquid nitrogen and lyophilized using a freeze dryer (Edwards Ltd., Crawley, England).

### NMR measurements

NMR sample preparation and measurements were performed according to a protocol that was published previously^26^. Briefly, 500 μl of methanol-*d*_*4*_ were added to freeze-dried samples, and the resultant mixtures were vortexed for 10 sec and sonicated for 20 min at 42 kHz using an Ultrasonicator 5510E-MT (Branson, Danbury, CT, USA), followed by centrifugation at 16,000x *g* at room temperature for 5 min. The supernatant (300 μl) was transferred to a 3 mm micro NMR tube and analyzed. The ^1^H NMR spectra were recorded at 25 °C on a 600 MHz Bruker DMX-600 spectrometer (Bruker, Karlsruhe, Germany) operating at a proton NMR frequency of 600.13 MHz. Deuterated methanol was used as the internal lock. Each ^1^H NMR spectrum consisted of 128 scans using the following parameters: 0.16 Hz/point, pulse width (PW) = 30 (11.3 ls) and relaxation delay (RD) = 1.5 s. Free induction decays (FIDs) were Fourier transformed with a line broadening (LB) = 0.3 Hz. The resulting spectra were manually phased and baseline corrected, and calibrated to methanol at 3.30 ppm, using XWIN NMR (version 3.5, Bruker).

### UHPLC-TOF mass spectrometry

The UHPLC-TOF-MS analyses were performed on an Ultimate 3000 UHPLC system (Thermoscientific, USA) coupled to a micro-ToF-2Q mass spectrometer from Bruker Daltonics (Bremen, Germany) with an electrospray (ESI) interface^48^. In separate runs, detection was achieved in both positive and negative ion modes. The *m/z* range was set to be 100–1000 and the ESI conditions were as follows: capillary voltage of 3500 V, source temperature of 250 °C, desolvation temperature of 250 °C, dry gas low of 10.0 l/min and the nebulizer at 2.0 Bar. For positive ionization, all conditions were the same and the capillary voltage was 4000 V. Internal calibration was performed using a 10 mM sodium formate solution from Sigma–Aldrich (Steinheim, Germany). Samples of 3 μl were injected onto a Kinetex C18, 150 × 2.0 mm column, packed with 1.7 μm particles (Phenomenex, USA), and eluted in gradient mode at a flow rate of 0.3 ml/min with the following solvent system: (A) 0.1% formic acid (v/v) in water; (B) 0.1% (v/v) formic acid in acetonitrile. Analysis began with a gradient of 5% to 90% B to in 19.5 min, followed by an isocratic step of 90% B for 1 min and a re-equilibration of 1 minute with 5% B. The total run time was 21.5 min. The temperature was maintained at 30 °C.

### Multivariate Data analysis

The ^1^H NMR data files were processed as described^26^,^49^. The ^1^H NMR spectra were converted to an ASCII file using AMIX software (Bruker Biospin GmbH), with total intensity scaling. Bucketing or binning was performed and the spectral data were reduced to include regions of equal width (0.04 ppm) equivalent to the region of *δ* 0.30–10.02. The regions of *δ* 4.85 – 4.95 and *δ* 3.25 – 3.35 were removed in the analysis because of the remnant signals of the solvents, HDO and CD_3_OD, respectively. Principal component analysis (PCA) was performed with the SIMCA-P+ software (Version 13.0, Umetrics, Umeå, Sweden). Pareto-scaling method was used for PCA.

## Additional Information

**How to cite this article**: Wu, C. *et al.* Expanding the chemical space for natural products by *Aspergillus-Streptomyces* co-cultivation and biotransformation. *Sci. Rep.* **5**, 10868; doi: 10.1038/srep10868 (2015).

## Supplementary Material

Supplementary Information

## Figures and Tables

**Table 1:** Spectral data assignments for the compounds displayed in Fig. 3. All compounds were summarized according to their corresponding producers, namely *S. coelicolor* monoculture, *A. niger* monoculture, and referred co-culture. Compound identification was based on ^1^H NMR and/or high resolution mass spectrometry, and compared with literature. Proton coupling constants (*J* in Hz) are given in parentheses.

**Producer**	**Compounds**	**Molecular Formula**	**Characteristic** ^**1**^**H NMR Chemical shifts**	**Exact Mass**	**Experimental high resolution mass**	**Reference**
***S. coelicolor***	Undecylprodigiosin	C_25_H_35_N_3_O	7.32 (dd, *J *= 2.4, 1.2); 6.37 (dd, *J *= 4.2, 1.2); 6.18 (d, *J *= 4.2); 2.78 (m)	393.2780	394.2854 [M + H]^+^	31
	Actinorhodin	C_32_H_26_O_14_	7.38 (s)	634.1323	657.3169 [M + Na]^+^; 633.1256 [M – H]^–^	30
***A. niger***	Carbonarones A	C_13_H_11_NO_3_	8.88 (s); 7.24 (d, *J *= 8.4); 7.37 (m); 7.31 (m); 6.38 (s); 3.88 (s)	229.0739	230.0809 [M + H]^+^; 252.0630 [M + Na]^+^; 269.1353 [M + K]^+^;	32
	Fumonisin B2	C_34_H_59_NO_14_		705.3936	706.4009 [M + H]^+^	34
	Fumonisin B4	C_34_H_59_NO_13_		689.3986	690.4063 [M + H]^+^; 688.3917 [M – H]^–^	33
	Aurasperone B	C_32_H_30_O_12_	6.64 (d, *J *= 2.4); 3.93 (s); 3.72 (s); 3.60 (s); 3.13 (d, *J *= 17.4); 3.12 (d, *J *= 17.4)	606.1737	607.1808 [M + H]^+^; 629.1605 [M + Na]^+^; 605.1665 [M – H]^–^	33
**Co-culture**	Cyclo-(Phe-Phe)	C_18_H_18_N_2_O_2_	7.33 (t, *J *= 7.8); 7.25 (m); 7.10 (brd, *J* = 7.8); 4.08 (dd, *J *= 7.2, 4.2); 2.78 (dd, *J *= 13.2, 4.2); 2.14 (dd, *J *= 13.2, 7.2)	294.1368	295.1429 [M + H]^+^; 317.1238 [M + Na]^+^	35
	Cyclo-(Phe-Tyr)	C_18_H_18_N_2_O_3_	7.33 (t, *J *= 7.8); 7.25 (m); 7.10 (brd, *J *= 7.8); 6.88 (d, *J *= 8.4); 6.68 (d, *J *= 8.4)	310.1317	311.1379 [M + H]^+^; 333.1185 [M + Na]^+^	35
	Phenylacetic acid	C_8_H_8_O_2_	3.58 (s); 6.77 (m); 7.09 (m)	136.0524	135.0442 [M − H]^–^	37
	2-hydroxyphenylacetic	C_8_H_8_O_3_	3.47 (s)	152.0473	153.0551 [M + H]^+^	40
	Furan-2-carboxylic acid	C_5_H_4_O_3_	7.61 (dd, *J *= 1.8, 1.2); 7.06 (dd, *J *= 3.6, 0.6); 6.51 (dd, *J *= 3.6, 1.8)	112.0160	135.0413 [M + Na]^+^; 111.0090 [M − H]^–^	38

**Table 2:** ^1^H and ^13^C NMR data assignment for new compound (2*E*,4*E*)-3-(2-carboxy-1-hydroxyethyl)-2,4-hexadienedioxic acid (**1**) in CD_3_OD. Proton coupling constants (*J* in Hz) are given in parentheses. ^1^H NMR and ^13^C NMR spectra were recorded at 600 MHz. All chemical shift assignments were done on the basis of 1D- and 2D -NMR techniques.

**NO.**	***δ***_***C***_	***δ***_***H***_ (***J*** **in Hz)**	**HMBC (**^**1**^**H→**^**13**^**C)**	**COSY (**^**1**^**H→**^**1**^**H)**
1	172.4			
2	120.6	6.42 (d, *J* = 0.6)	C-1, C-4, C-1'	H-1'
3	162.5			
4	132.0	7.47 (dt, *J *= 16.2, 0.6)	C-1’, C-2, C-6, C-5, C-3	H-5, H-1'
5	128.5	6.42 (d, *J *= 16.2)	C-6, C-3, C-4	H-4
6	167.1			
1'	79.0	5.68 (dddd, *J *= 7.2, 3.6, 1.8, 0.6)		H-2, H-4, H-2'
2'	37.6	3.07 (dd, *J *= 16.8, 3.6); 2.58 (dd, *J *= 16.8, 3.6)	C-3’, C-3	H-1'
3'	171.1			
