# Supplementary material for: Expanding the chemical space for natural products by Aspergillus-Streptomyces co-cultivation and biotransformation
Source: Sci Rep. 2015 Jun 4;5:10868. doi: 10.1038/srep10868 (PMC4455117; doi:10.1038/srep10868)
Supplement: Supplementary Information [file srep10868-s1.pdf]

Expanding the chemical space for natural products by *Aspergillus-Streptomyces* co-cultivation and biotransformation

Changsheng Wu [1,2], Boris Zacchetti [1], Arthur F.J. Ram [1], Gilles P. van Wezel [1,#], Dennis Claessen [1,*] and Young Hae Choi [2,*,^]

[1] *Molecular Biotechnology, Institute of Biology, Leiden University, Sylviusweg 72, 2333 BE, The Netherlands;*

[2] *Natural Products Laboratory, Institute of Biology, Leiden University, Sylviusweg 72, 2333 BE, The Netherlands*

Author for correspondence. Tel +31 71 5274310, E-mail: g.wezel@biology.leidenuniv.nl;

^ Additional author for correspondence. Tel +31 71 5274510, E-mail: y.choi@chem.leidenuniv.nl

* these authors contributed equally to the work

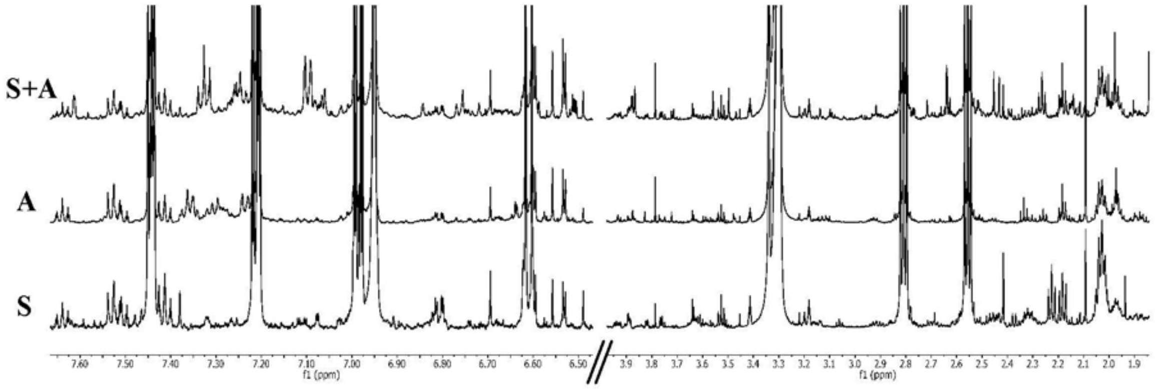

Figure S1. ^{1}H NMR spectra in the region of δ 6.50—7.60, and 1.90—3.90 of *Streptomyces coelicolor* single culture (**S**), *Aspergillus niger* single culture (**A**), and coculture of *Streptomyces coelicolor* with *Aspergillus niger* (**S+A**). Major discriminators for PCA separation (Figure 2) are summarized in Table 1.

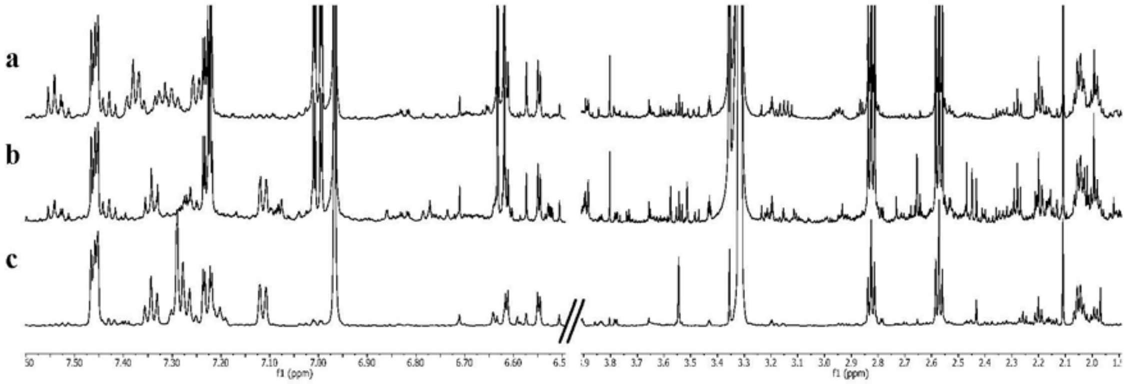

Figure S2. ^{1}H NMR spectra in the region of *δ* 6.50—7.60 and 1.90—3.90 of *Aspergillus niger* single culture (a), *Aspergillus niger* cocultured with *Streptomyces coelicolor* (b), and *Aspergillus niger* cultured in a cell-free extract of *Streptomyces coelicolor* (c). Cell-free extract of *S. coelicolor* was sufficient for eliciting *Aspergillus niger* to produce cyclo-(Phe-Phe) and phenylacetic acid

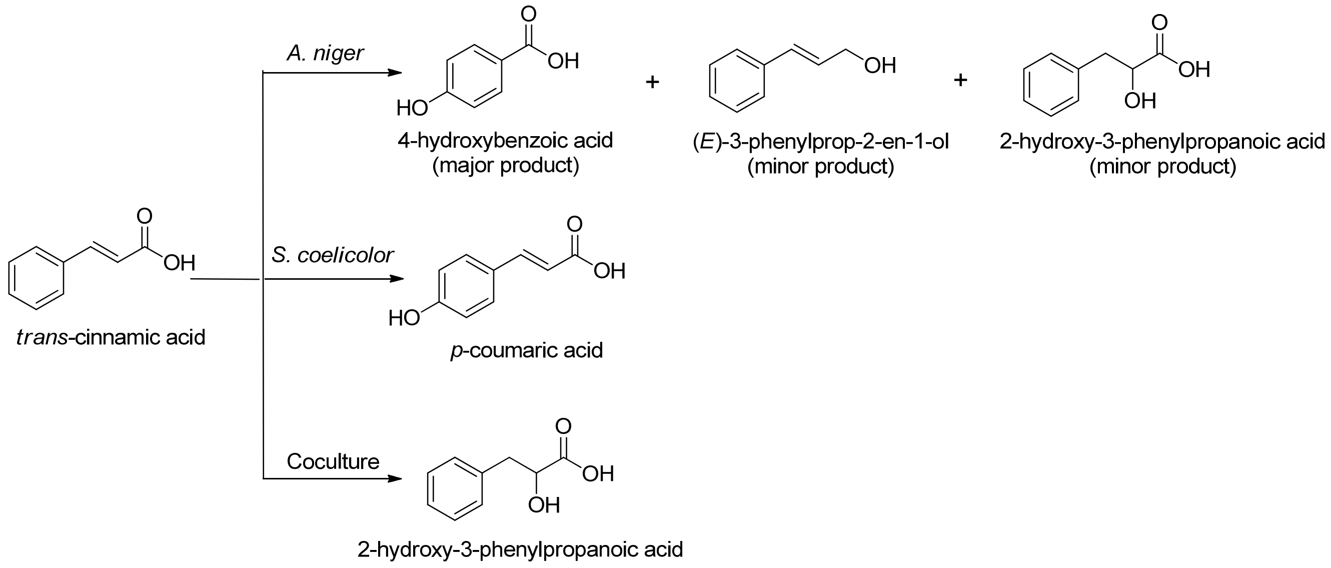

Figure S3. Biotransformation products of *trans*-cinnamic acid by *S. coelicolor* monoculture, *A. niger* monoculture and their coculture.

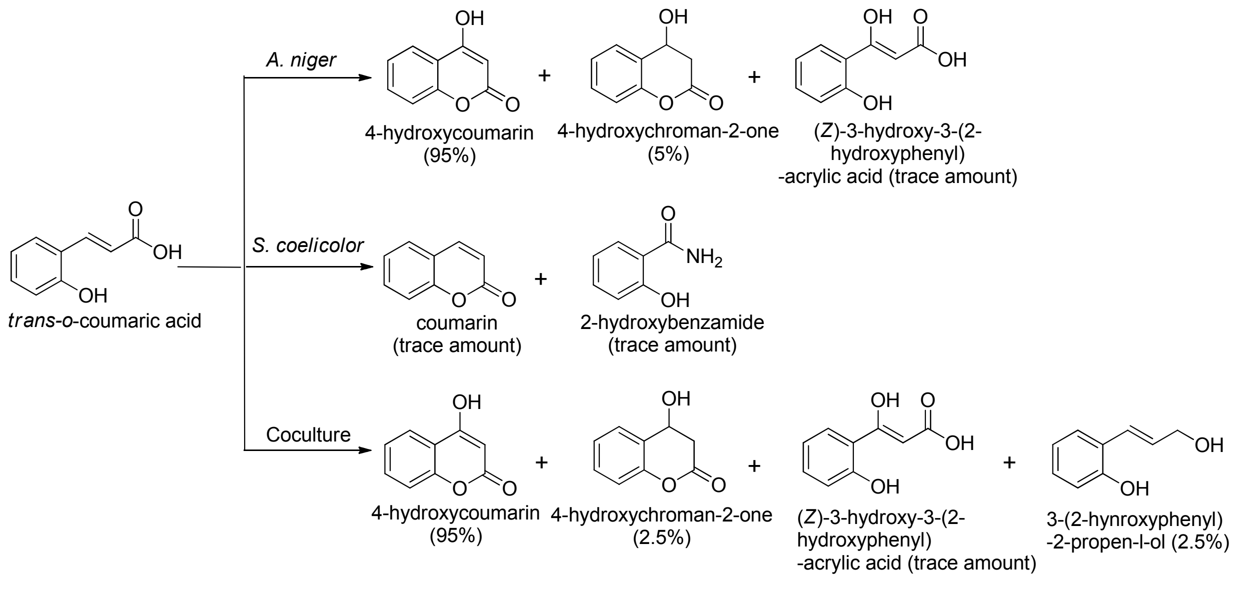

Figure S4. biotransformation products of *trans*-*o*-coumaric acid by *S. coelicolor* monoculture, *A. niger* monoculture and their coculture.

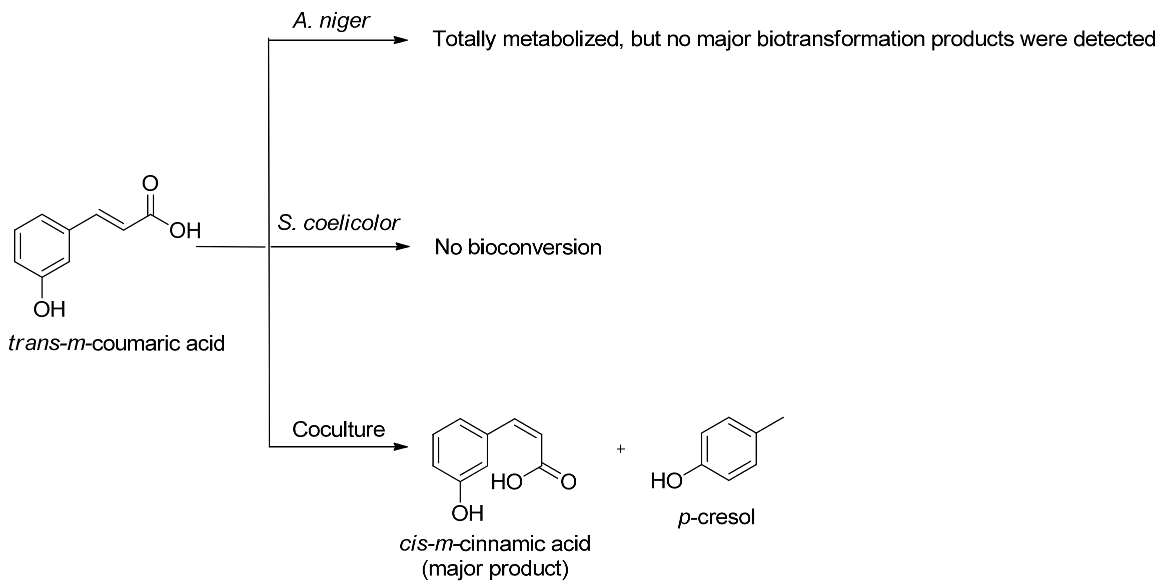

Figure S5. biotransformation products of *trans-m*-coumaric acid by *S. coelicolor* monoculture, *A. niger* monoculture and their coculture.

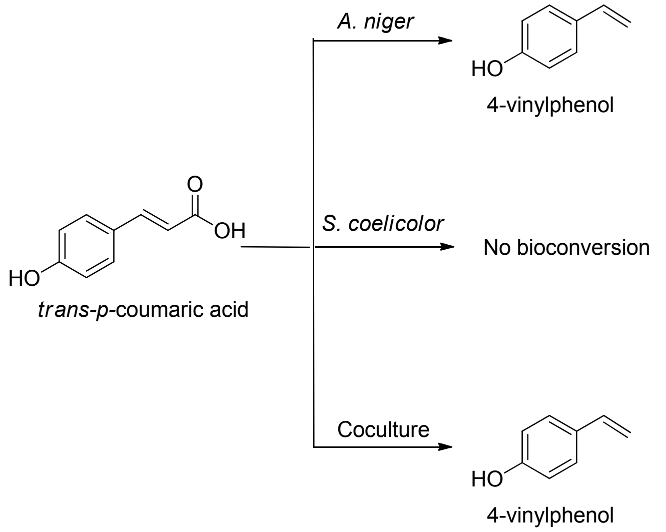

Figure S6. biotransformation products of *trans-p*-coumaric acid by *S. coelicolor* monoculture, *A. niger* monoculture and their coculture.

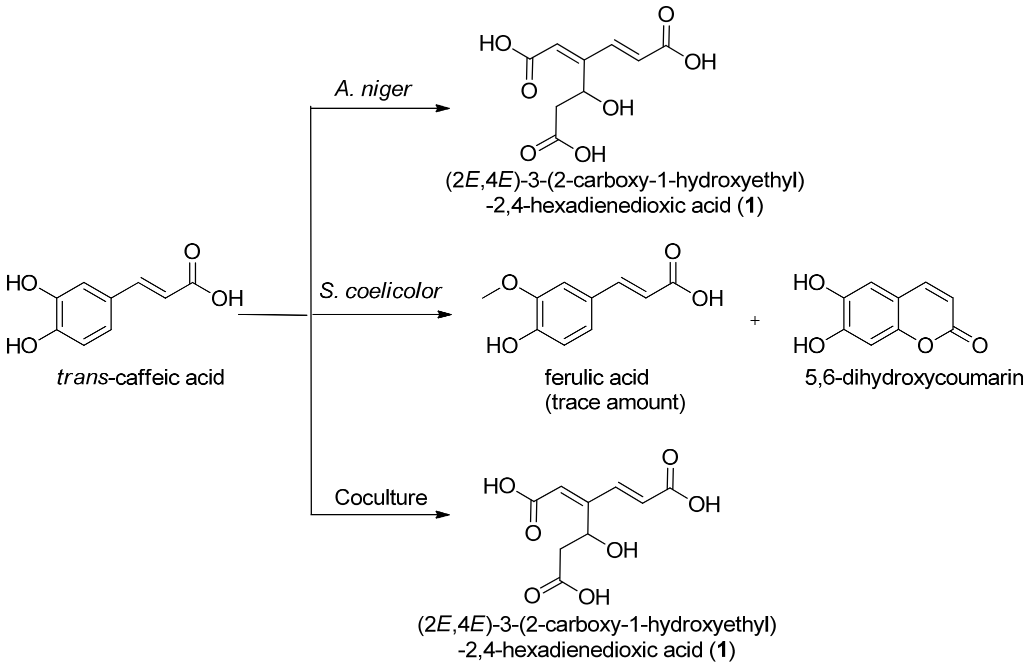

Figure S7. biotransformation products of *trans*-caffeic acid by *S. coelicolor* monoculture, *A. niger* monoculture, and coculture.

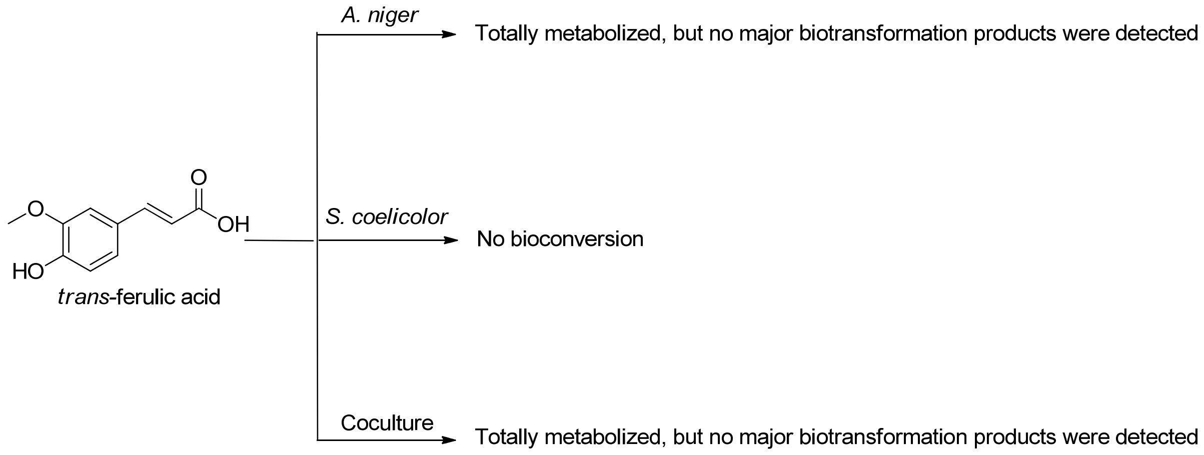

Figure S8. biotransformation products of *trans*-ferulic acid by *S. coelicolor* monoculture, *A. niger* monoculture, and coculture.

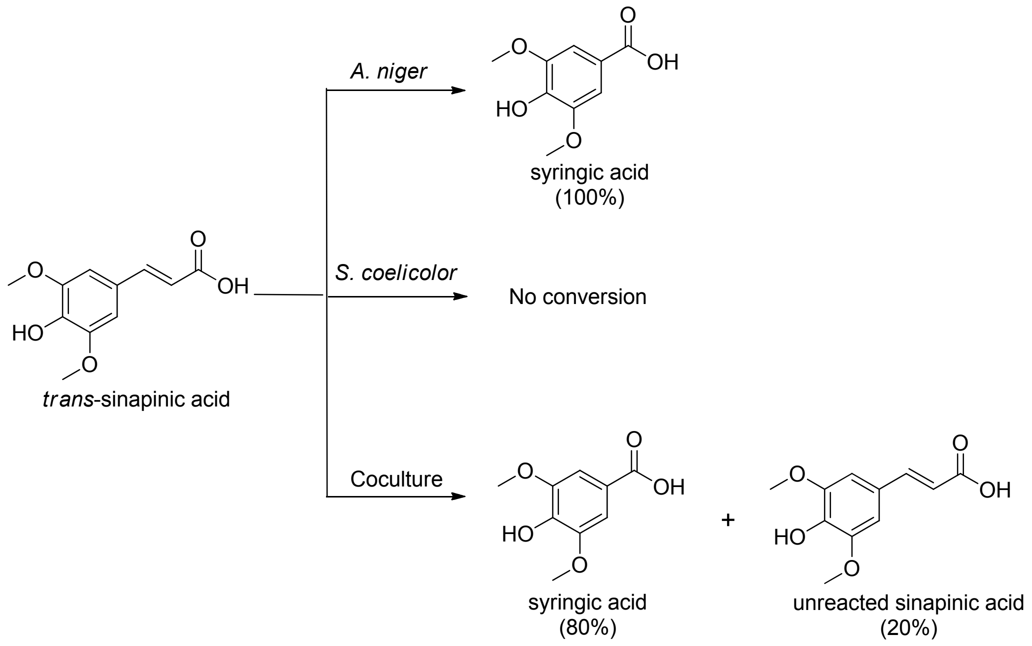

Figure S9. biotransformation products of *trans*-sinapinic acid by *S. coelicolor* monoculture, *A. niger* monoculture and their coculture.

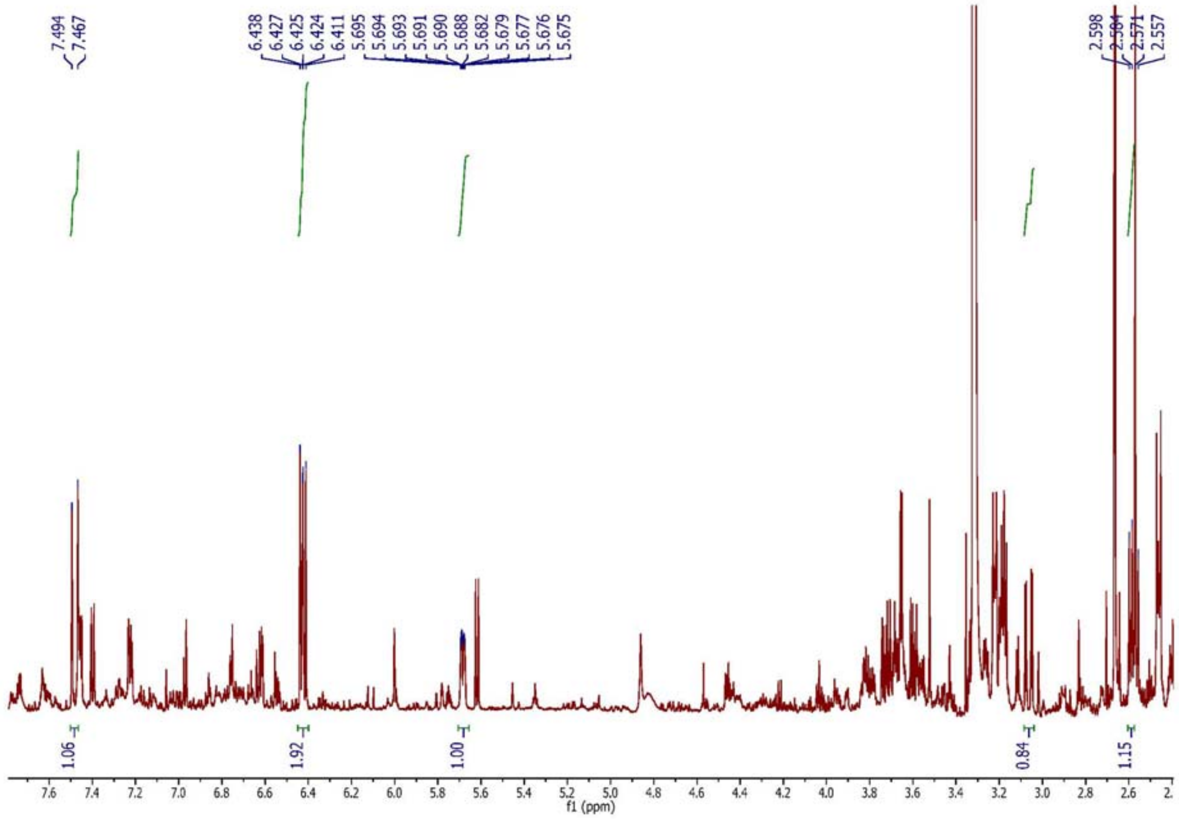

Figure S10. ^{1}H NMR spectrum of caffeic acid biotransformation by coculture of *S. coelicolor* with *A. niger*. Integrated signals belong to (2*E*,4*E*)-3-(2-carboxy-1-hydroxyethyl)-2,4-hexadienedioxic acid (**1**).

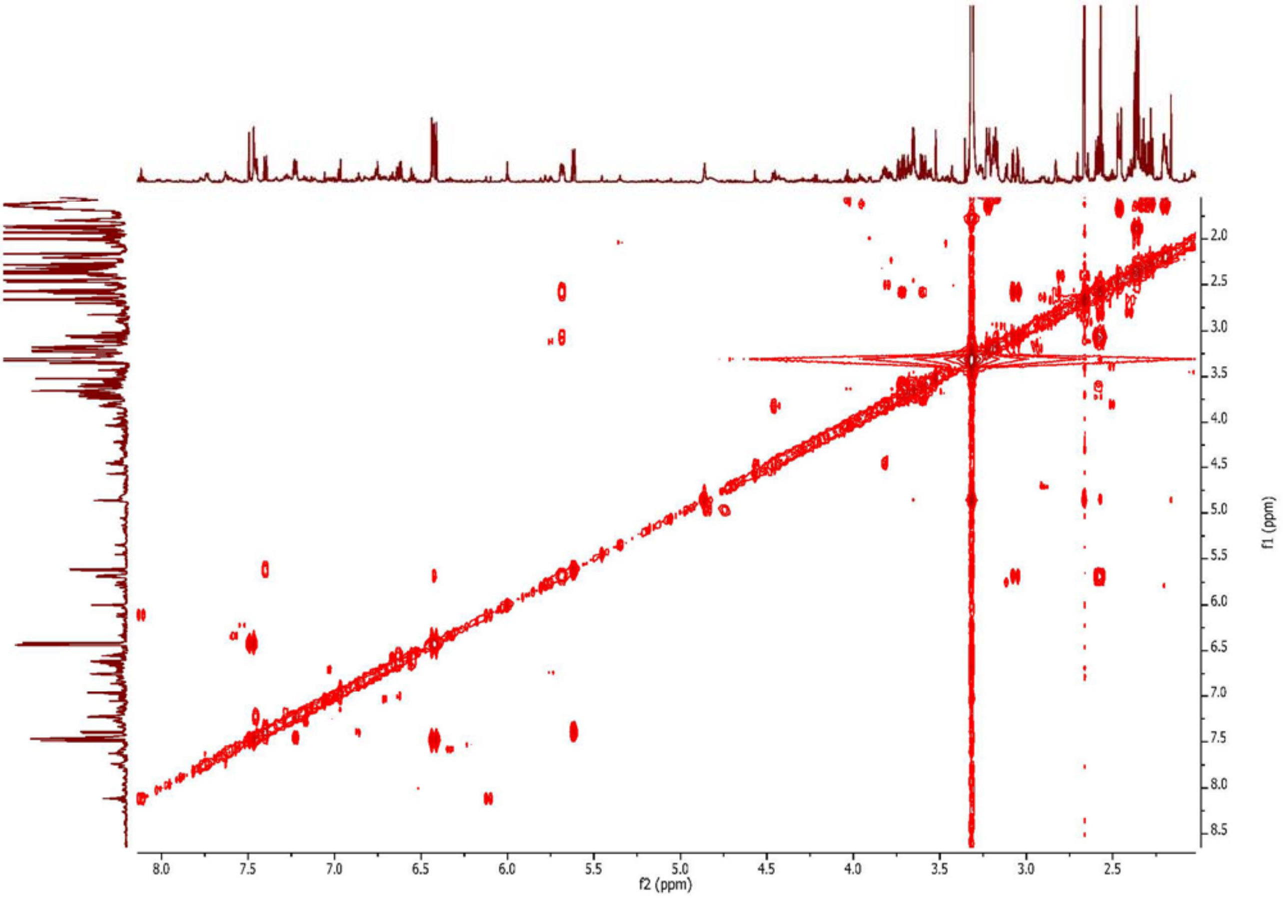

Figure S11. ^{1}H-^{1}H COSY of caffeic acid biotransformation by coculture of *S. coelicolor* with *A. niger*.

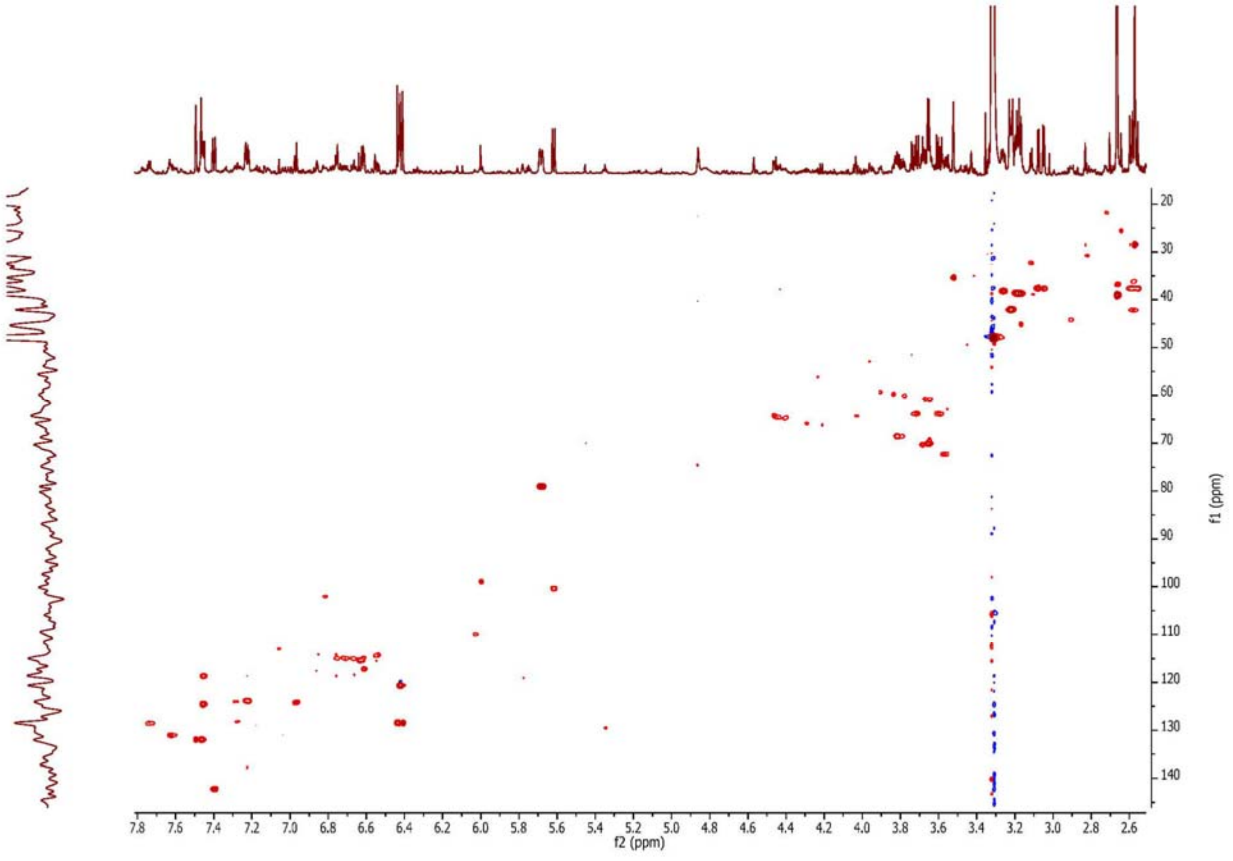

Figure S12. HSQC spectrum of caffeic acid biotransformation by coculture of *S. coelicolor* with *A. niger*.

.

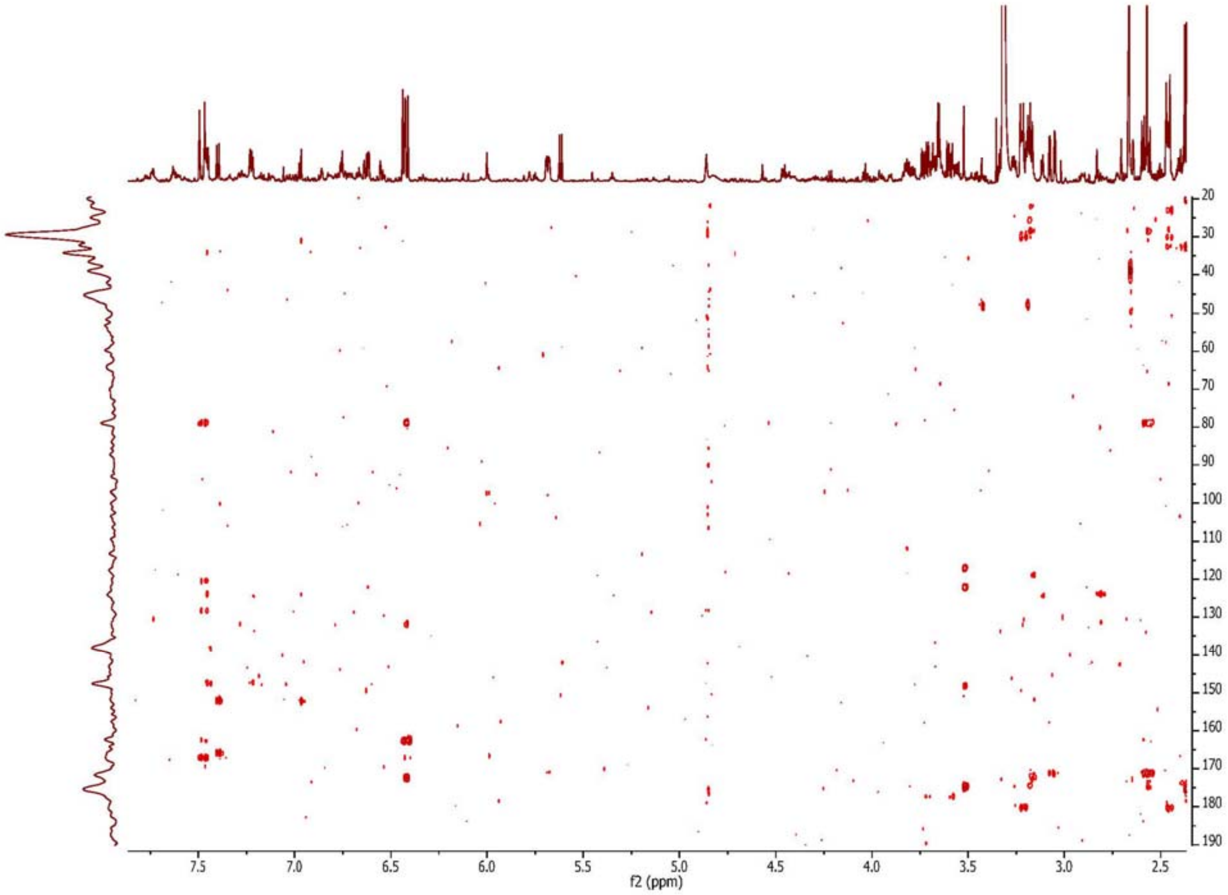

Figure S13. HMBC spectrum of caffeic acid biotransformation by coculture of *S. coelicolor* with *A. niger*.

.

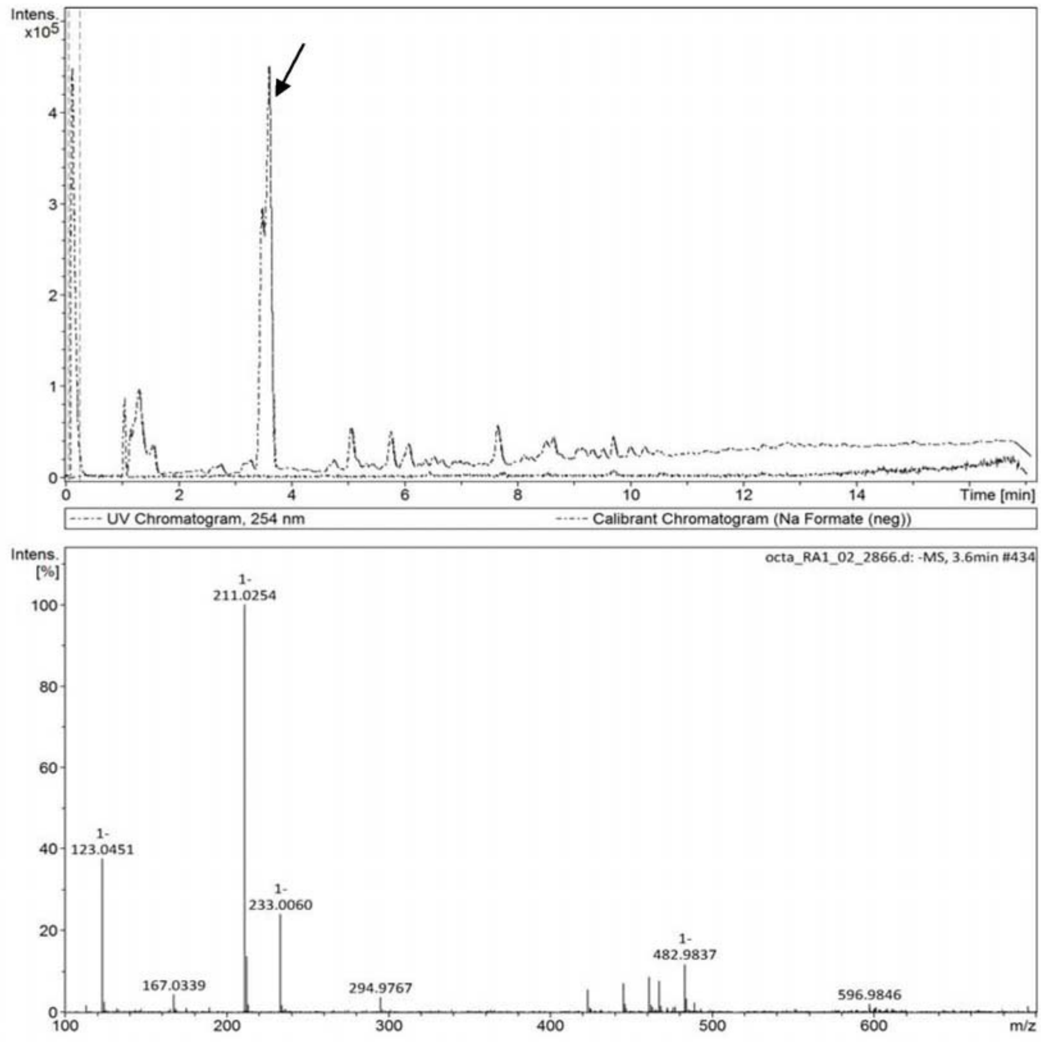

Figure S14. U(H)PLC-Q-TOF analysis of caffeic acid biotransformation by coculture of *S. coelicolor* with *A. niger*. Arrow points at the MS peak at 211.0254.

Table S1. ^{1}H NMR and HRMS assignment for the biotransformation products of *trans*-cinnamic acid by *S. coelicolor* monoculture, *A. niger* monoculture, and their coculture.

Origin	Compounds	Characteristic ^{1}H NMR Chemical shifts	UHPLC-Q-TOF-MS
**Substrate**	*trans*-cinnamic acid	7.68 (d, *J* = 16.2 Hz); 6.48 (d, *J* = 16.2 Hz); 7.59 (m); 7.41 (m)	149.0604 $[M + H]^+$; 131.0507 $[M - H_2O + H]^+$; 147.0451 $[M - H]^-$
S. coelicolor	*p*-coumaric acid	6.28 (d, *J* = 16.2 Hz); 6.81 (d, *J* = 8.4 Hz)	165.0538 $[M + H]^+$; 147.0438 $[M - H_2O + H]^+$; 163.0397 $[M - H_2O - H]^+$;
A. niger	4-hydroxybenzoic acid	7.88 (d, *J* = 9.0 Hz); 6.82 (d, *J* =9.0 Hz);	139.0390 $[M + H]^+$; 121.0280 $[M - H_2O + H]^+$; 137.0238 $[M - H]^-$
	(*E*)-3-phenylprop-2-en-1-ol	7.41 (m); 7.39 (m); 6.37 (dt, *J* = 16.2, 6.0 Hz); 4.23 (dd, *J* = 6.0, 1.8 Hz)	117.0707 $[M - H_2O + H]^+$
	2-hydroxy-3-phenylpropanoic acid	7.27 (m); 4.31 (dd, *J* = 7.8, 4.2 Hz); 3.10 (dd, *J* = 7.8, 4.2 Hz)	165.0553 $[M - H]^-$; 147.0461 $[M - H_2O + H]^+$
**Coculture**	2-hydroxy-3-phenylpropanoic acid	7.27 (m); 4.31 (dd, *J* = 7.8, 4.2 Hz); 3.10 (dd, *J* = 7.8, 4.2 Hz)	165.0553 $[M - H]^-$; 147.0461 $[M - H_2O + H]^+$

Table S2. ^{1}H NMR and HRMS assignment for the biotransformation products of *trans-o*-coumaric acid by *S. coelicolor* monoculture, *A. niger* monoculture and their coculture.

Origin	Compounds	Characteristic ^{1}H NMR Chemical shifts	UHPLC-Q-TOF-MS
**Substrate**	*trans-o*-coumaric acid	7.97 (d, *J* = 16.2 Hz); 6.55 (d, *J* = 16.2 Hz); 7.48 (dd, *J* =8.4, 1.8 Hz);7.21 (td, *J* =8.4, 1.8 Hz); 6.84 (td, *J* =8.4, 1.8 Hz); 6.85 (dd, *J* =8.4, 1.8 Hz);	165.0549 $[M + H]^+$; 187.0358 $[M + Na]^+$; 147.0441 $[M – H_2O + H]^+$; 163.0408 $[M – H]^–$
S. coelicolor	coumarin	6.43 (d, *J* = 9.6 Hz); 7.60 (td, *J* =8.4, 1.8 Hz); 7.29 (m)	147.0431 $[M + H]^+$
	2-hydroxybenzamide	7.81 (dd, *J* =8.4, 1.8 Hz); 7.23 (td, *J* =8.4, 1.8 Hz); 6.73 (dd, *J* =8.4, 1.8 Hz); 6.57 (td, *J* =8.4, 1.8 Hz)	138.0548 $[M + H]^+$; 120.0441 $[M – H_2O +H]^+$; 136.0405 $[M – H]^–$
	(E)-methyl 3-(2-hydroxyphenyl)acrylate	3.60 (s)	179.0703 $[M + H]^+$; 177.0552 $[M – H]^–$
A. niger	4-dihydroxycoumarin	7.91 (dd, *J* =7.8, 1.8 Hz); 7.64 (td, *J* =8.4, 1.2 Hz); 7.35 (td, *J* =8.4, 1.2 Hz); 7.34 (dd, *J* =7.8, 1.8 Hz); 5.65 (s)	163.0375 $[M + H]^+$; 185.0207 $[M + Na]^+$; 161.0274 $[M – H]^–$
	4-hydroxychroman-2-one	7.22 (dd, *J* =8.4, 2.4 Hz); 7.08 (td, *J* =7.8, 1.2 Hz); 6.82 (td, *J* =7.8, 1.2 Hz); 6.76 (dd, *J* =8.4, 1.2 Hz); 5.39 (dd, *J* =9.6, 3.6 Hz); 2.79 (dd, *J* =15.6, 3.6 Hz); 2.59 (dd, *J* = 15.6, 9.6 Hz)	165.0547 $[M + H]^+$; 147.0439 $[M – H_2O +H]^+$; 163.0396 $[M – H]^–$
	(Z)-3-hydroxy-3-(2-hydroxyphenyl)acrylic acid	7.96 (dd, *J* =7.8, 1.8 Hz); 7.52 (td, *J* =8.4, 1.2 Hz); 7.27 (td, *J* =8.4, 1.2 Hz); 7.26 (dd, *J* =7.8, 1.8 Hz); 5.90 (s)	181.0490 $[M + H]^+$; 203.0331 $[M + Na]^+$; 163.0391 $[M – H_2O +H]^+$; 179.0346 $[M – H]^–$;
	2-allylphenol	6.33 (m); 5.15 (dt, *J* = 10.2, 1.8 Hz); 5.12 (dt, *J* = 16.8, 1.8 Hz);	135.0796 $[M + H]^+$;
**Coculture**	4-dihydroxycoumarin	7.91 (dd, *J* =7.8, 1.8 Hz); 7.64 (td, *J* =8.4, 1.2 Hz); 7.35 (td, *J* =8.4, 1.2 Hz); 7.34 (dd, *J* =7.8, 1.8 Hz); 5.65 (s)	163.0375 $[M + H]^+$; 185.0207 $[M + Na]^+$; 161.0274 $[M – H]^–$
	4-hydroxychroman-2-one	7.22 (dd, *J* =8.4, 2.4 Hz); 7.08 (td, *J* =7.8, 1.2 Hz); 6.82 (td, *J* =7.8, 1.2 Hz); 6.76 (dd, *J* =8.4, 1.2 Hz); 5.39 (dd, *J* =9.6, 3.6 Hz); 2.79 (dd, *J* =15.6, 3.6 Hz); 2.59 (dd, *J* = 15.6, 9.6 Hz)	165.0547 $[M + H]^+$; 147.0439 $[M – H_2O +H]^+$; 163.0396 $[M – H]^–$
	3-(2-hynroxyphenyl)-2-propen-l-ol	7.36 (td, *J* = 7.8, 1.2 Hz); 7.04 (td, *J* = 7.8, 1.8 Hz); 6.76 (dd, *J* = 7.8, 1.2 Hz); 6.88 (dt, *J* = 16.2, 1.8 Hz); 6.36 (dt, *J* = 16.2, 6.0 Hz); 4.22 (dd, *J* = 6.0, 1.8 Hz)	133.0649 $[M – H_2O +H]^+$; 149.0615 $[M – H]^–$; 131.0502 $[M – H_2O – H]^–$
	(Z)-3-hydroxy-3-(2-hydroxyphenyl)acrylic acid	7.96 (dd, *J* =7.8, 1.8 Hz); 7.52 (td, *J* =8.4, 1.2 Hz); 7.27 (td, *J* =8.4, 1.2 Hz); 7.26 (dd, *J* =7.8, 1.8 Hz)); 5.90 (s)	181.0490 $[M + H]^+$; 203.0331 $[M + Na]^+$; 163.0391 $[M – H_2O +H]^+$; 179.0346 $[M – H]^–$;
	2-allylphenol	6.33 (m); 5.15 (dt, *J* = 10.2, 1.8 Hz); 5.12 (dt, *J* = 16.8, 1.8 Hz);	135.0796 $[M + H]^+$;

Table S3. ^{1}H NMR and HRMS assignment for the biotransformation products of *trans*-*m*-coumaric acid by *S. coelicolor* monoculture, *A. niger* monoculture and their coculture.

Origin	Compounds	Characteristic ^{1}H NMR Chemical shifts	UHPLC-Q-TOF-MS
**Substrate**	*trans*-*m*-coumaric acid	7.59 (d, *J* = 16.2 Hz); 6.41 (d, *J* = 16.2 Hz); 7.22 (t, *J* = 7.8 Hz); 7.05 (brd, *J* = 7.2 Hz); 7.00 (t, *J* = 1.8 Hz); 6.84 (ddd, *J* = 8.4, 2.4, 1.2 Hz)	165.0547 $[M + H]^+$; 187.0361 $[M + Na]^+$; 147.0442 $[M - H_2O + H]^+$; 163.0417 $[M - H]^-$
S. coelicolor	No products		
A. niger	No major biotransformation products		
**Coculture**	*cis*-*m*-coumaric acid	6.87 (d, *J* = 12.6 Hz); 5.93 (d, *J* = 12.6 Hz); 7.15 (t, *J* = 7.8 Hz); 7.06 (t, *J* = 1.8 Hz); 7.01 (ddd, *J* = 8.4, 2.4, 1.2 Hz)	147.0432 $[M - H_2O + H]^+$; 163.0405 $[M - H]^-$;
	p-cresol	6.73 (d, *J* = 8.4 Hz); 7.09 (d, *J* = 8.4 Hz)	107.0501 $[M - H]^-$

Table S4. ^1H NMR and HRMS assignment for the biotransformation products of *trans*-*p*-coumaric acid by *S. coelicolor* monoculture, *A. niger* monoculture and their coculture.

Origin	Compounds	Characteristic ^1H NMR Chemical shifts	UHPLC-Q-TOF-MS
**Substrate**	*trans*-*p*-coumaric acid	7.61 (d, *J* = 16.2 Hz); 6.29 (d, *J* = 16.2 Hz); 7.45 (d, *J* = 8.4 Hz); 6.81 (d, *J* = 8.4 Hz)	165.0548 $[M + H]^+$; 187.0361 $[M + Na]^+$; 147.0446 $[M – H_2O + H]^+$; 163.0417 $[M – H]^–$
S. coelicolor	No products		
A. niger	4-vinylphenol	6.63 (dd, *J* = 17.4, 10.8 Hz); 5.56 (dd, *J* = 17.4, 1.2 Hz); 5.03 (dd, *J* = 10.8, 1.2 Hz); 6.73 (d, *J* = 8.4 Hz); 7.26 (d, *J* = 8.4 Hz)	121.0649 $[M + H]^+$; 119.0497 $[M – H]^–$
**Coculture**	4-vinylphenol	6.63 (dd, *J* = 17.4, 10.8 Hz); 5.56 (dd, *J* = 17.4, 1.2 Hz); 5.03 (dd, *J* = 10.8, 1.2 Hz); 6.73 (d, *J* = 8.4 Hz); 7.26 (d, *J* = 8.4 Hz)	121.0649 $[M + H]^+$; 119.0497 $[M – H]^–$

Table S5. ^{1}H NMR and HRMS assignment for the biotransformation products of *trans*-caffeic acid by *S. coelicolor* monoculture, *A. niger* monoculture and their coculture.

Origin	Compounds	Characteristic ^{1}H NMR Chemical shifts	UHPLC-Q-TOF-MS
**Substrate**	*trans*-caffeic acid	7.53 (d, *J* = 16.2 Hz); 6.22 (d, *J* = 16.2 Hz); 7.04 (d, *J* = 1.8 Hz); 6.94 (dd, *J* = 8.4, 1.8 Hz); 6.79 (d, *J* = 8.4 Hz)	181.0492 $[M + H]^+$; 203.0312 $[M + Na]^+$; 163.0393 $[M - H_2O + H]^+$; 179.0374 $[M - H]^-$
S. coelicolor	*trans*-ferulic acid	7.60 (d, *J* = 16.2 Hz); 6.32 (d, *J* = 16.2 Hz); 7.18 (d, *J* = 1.8 Hz); 7.07 (dd, *J* = 8.4, 1.8 Hz); 6.82 (d, *J* = 8.4 Hz); 3.90 (s)	195.0657 $[M + H]^+$; 177.0540 $[M - H_2O + H]^+$; 193.0514 $[M - H]^-$
	5,6-dihydroxycoumarin	7.79 (d, *J* = 9.6 Hz); 6.19 (d, *J* = 9.6 Hz); 6.76 (s)	179.0335 $[M + H]^+$; 177.0200 $[M - H]^-$
A. niger	(2*E*,4*E*)-3-(2-carboxy-1-hydroxyethyl) -2,4-hexadienedioxic acid (**1**)	see Table 2	211.0254 $[M - H_2O - H]^-$
**Coculture**	(2*E*,4*E*)-3-(2-carboxy-1-hydroxyethyl) -2,4-hexadienedioxic acid (**1**)	see Table 2	211.0254 $[M - H_2O - H]^-$

Table S6. ^{1}H NMR and HRMS assignment for the biotransformation products of *trans*-ferulic acid by *S. coelicolor* monoculture, *A. niger* monoculture and their coculture.

Origin	Compounds	Characteristic ^{1}H NMR Chemical shifts	UHPLC-Q-TOF-MS
**Substrate**	*trans*-ferulic acid	7.60 (d, *J* = 16.2 Hz); 6.32 (d, *J* = 16.2 Hz); 7.18 (d, *J* = 2.4 Hz); 7.07 (dd, *J* = 8.4, 2.4 Hz); 6.82 (d, *J* = 8.4 Hz); 3.90 (s)	195.0661 $[M + H]^+$; 217.0447 $[M + Na]^+$; 177.0563 $[M - H_2O + H]^+$; 193.0511 $[M - H]^-$
S. coelicolor	No products		
A. niger	No products		
**Coculture**	No products		

Table S7. ^{1}H NMR and HRMS assignment for the biotransformation products of *trans*-sinapinic acid by *S. coelicolor* monoculture, *A. niger* monoculture and their coculture.

Origin	Compounds	Characteristic ^{1}H NMR Chemical shifts	UHPLC-Q-TOF-MS
Substrate	*trans*-sinapinic acid	7.60 (d, *J* = 16.2 Hz); 6.34 (d, *J* = 16.2 Hz); 6.90 (s)	225.0779 $[M + H]^+$; 247.0590 $[M + Na]^+$; 207.0683 $[M – H_2O + H]^+$; 223.0633 $[M – H]^–$
S. coelicolor	No conversion		
A. coelicolor	4-hydroxy-3,5-dimethoxybenzoic acid	7.33 (s); 3.89 (s)	199.0608 $[M + H]^+$; 221.0423 247.0590 $[M + Na]^+$; 181.0493 $[M – H_2O + H]^+$; 197.0468 $[M – H]^–$
Coculture	*trans*-sinapinic acid	7.60 (d, *J* = 16.2 Hz); 6.34 (d, *J* = 16.2 Hz); 6.90 (s)	225.0779 $[M + H]^+$; 247.0590 $[M + Na]^+$; 207.0683 $[M – H_2O + H]^+$; 223.0633 $[M – H]^–$
	4-hydroxy-3,5-dimethoxybenzoic acid	7.33 (s); 3.89 (s)	199.0608 $[M + H]^+$; 221.0423 247.0590 $[M + Na]^+$; 181.0493 $[M – H_2O + H]^+$; 197.0468 $[M – H]^–$
